# Gene aberration profile of tumors of adolescent and young adult females

**DOI:** 10.18632/oncotarget.23765

**Published:** 2017-12-29

**Authors:** Yasuyuki Kanke, Akihiko Shimomura, Motonobu Saito, Takayuki Honda, Kouya Shiraishi, Yoko Shimada, Reiko Watanabe, Hiroshi Yoshida, Masayuki Yoshida, Chikako Shimizu, Kazuaki Takahashi, Hirohiko Totsuka, Hideaki Ogiwara, Sou Hirose, Koji Kono, Kenji Tamura, Aikou Okamoto, Takayuki Kinoshita, Tomoyasu Kato, Takashi Kohno

**Affiliations:** ^1^ Division of Genome Biology, National Cancer Center Research Institute, Tokyo, Japan; ^2^ Department of Gastrointestinal Tract Surgery, Fukushima Medical University School of Medicine, Fukushima, Japan; ^3^ Department of Breast and Medical Oncology, National Cancer Center Hospital, Tokyo, Japan; ^4^ Pathology Division, Department of Pathology and Clinical Laboratories, National Cancer Center Hospital, Tokyo, Japan; ^5^ Department of Obstetrics and Gynecology, The Jikei University School of Medicine, Tokyo, Japan; ^6^ StaGen Co., Ltd., Tokyo, Japan; ^7^ Department of Breast Surgery, National Cancer Center Hospital, Tokyo, Japan; ^8^ Department of Gynecology, National Cancer Center Hospital, Tokyo, Japan

**Keywords:** adolescent and young adult (AYA), breast tumor, ovarian tumor, mutational signature, actionable gene

## Abstract

There has been little improvement in the prognosis for adolescent and young adult (AYA) tumor patients. Hence, there is an urgent need to understand the etiology of tumor development and identify actionable gene aberrations to improve prevention and therapy. Here, 76 sporadic tumors (48 breast, 22 ovarian, and six uterine) from 76 AYA females (age range, 25–39 years) were subjected to whole exome and RNA sequencing to determine their mutational signatures and actionable gene profiles. Two individuals with breast cancer (4.2% of cases) and one with ovarian cancer (5.3% of cases) carried germline *BRCA2* mutations. The two cases with breast tumors also each carried an additional deleterious germline mutation: one in *TP53* and the other in *CHEK2*. Mutational signature analysis of the 76 tumors indicated that spontaneous deamination of 5-methylcytosine and activity of the APOBEC cytidine deaminase protein family are major causes of mutagenesis. In addition, 18 breast or ovarian tumors (18/70, 26%), including the three cases with germline *BRCA2* mutations, exhibited a predominant “BRCAness” mutational signature, an indicator of functional *BRCA1/BRCA2* deficiency. Actionable aberrations and high tumor mutation burdens were detected in 24 breast (50%), 17 ovarian (77%), and five uterine (83%) tumor cases. Thus, mutational processes and aberrant genes in AYA tumors are largely shared with those identified in non-AYA tumors. The efficacy of molecular targeting and immune checkpoint inhibitory therapies should be explored for both AYA and non-AYA patients.

## INTRODUCTION

Nearly 68,000 adolescents and young adults (AYAs) aged 15 to 39 years were diagnosed with cancer in the US in 2002 [1], and females with breast, ovary, and uterine cancers constituted a large proportion of cases [2]. Since the prognosis for AYA patients with cancer has improved less than that for patients in the non-AYA age group [1, 3], better characterization of the properties of AYA cancers is urgently needed to facilitate understanding of the etiology of their early development and improve diagnosis and therapy.

The clinical and histopathological characteristics of tumors in AYA patients have been revealed by comparison with those of non-AYA patients in European/US populations [2]. Tumors of the breast in AYAs often lack expression of therapeutic targets, such as estrogen receptor (ER), progesterone receptor (PgR), and human epidermal growth factor receptor 2 (HER2) oncoprotein (*i.e*., they are of the triple-negative subtype), and have a poor prognosis [2, 4]. Malignant and borderline ovarian tumors are rare in adolescents compared with adults; however, they pose serious issues in that age group. Such ovarian tumors encompass a variety of subtypes; the most common are epithelial in origin; however, non-epithelial tumors, such as malignant germ cell and sex cord-stromal tumors, constitute a major fraction [5-8]. Cervical and endometrial carcinomas comprise a large proportion of AYA uterine tumors [9, 10].

Genome-wide mutation profiling of cancer genomes is a powerful method to identify actionable gene aberrations, and can facilitate elucidation of the mutagenic processes underlying the development of a variety of cancers [11-13]; however, few studies have focused on tumors in AYAs, and hence information on their associated gene aberrations is very limited. Approximately 2.5% of cancers in the Japanese population are diagnosed among the AYA age group, and, as in the US [2], breast, ovarian, and uterine cancers are the major types identified [14]. Here, we present gene aberration profiles of breast (*N* = 48), ovary (*N* = 22), and non-cervical uterine (*N* = 6) tumors from 76 AYA Japanese females at diagnosis.

## RESULTS

### Study cohort for genome-wide mutation profiling

The characteristics of the 76 sporadic AYA tumor cases studied here are presented in Table 1. The 48 breast tumors consisted of 47 carcinomas and one (2.1%) angiosarcoma. The histological and subtype distributions in the study cohort, including frequent luminal type carcinomas, were consistent with previous reports of breast tumors in Japanese AYAs [15]; however, they were different to the distributions among European/US patients, in whom both luminal and triple-negative tumors are common [16].

**Table 1:** Characteristics of the 76 female AYA tumor cases

		Breast tumor (*N*=48)			Ovarian tumor (*N*=22)			Uterine tumor (*N*=6)	
		*N*	%		*N*	%		*N*	%
Age	Mean (±SD)	36.6	(±2.9)		34.5	(±4.3)		34.5	(±3.9)
Stage	0	2	4.2		-	-		0	0
	I	16	33.3		12	54.5		1	16.7
	II	22	45.8		4	18.2		1	16.7
	III	7	14.6		6	27.2		4	66.7
	IV	0	0		0	0		0	0
	Unknown	1	2.1		0	0		0	0
Histology	DCIS	2	4.2	Serous (BM)	4 (2)	18.1	Endometrioid	5	83.3
	IDC	43	89.6	Mucinous (BM)	8 (2)	36.3	Carcinosarcoma	1	16.7
	ILC	1	2.1	Endometrioid (BM)	3 (1)	13.6			
	Mucinous	1	2.1	Clear cell	4	18.1			
	Angiosarcoma	1	2.1	Carcinosarcoma	1	4.5			
				Immature teratoma	1	4.5			
				Primitive neuroectodermal tumor and adenosquamous cell carcinoma	1	4.5			
Subtype	Luminal	36	75						
	Luminal HER2	3	6.3						
	HER2	2	4.2						
	Triple negative	6	12.5						
	Other	1	2.1						

SD, standard deviation; DCIS, ductal carcinoma in situ; IDC, invasive ductal carcinoma; ILC, invasive lobular carcinoma; BM, borderline malignancy.

The 22 ovarian tumors consisted of 14 carcinomas, five borderline tumors, and three others, while the six uterine tumors consisted of five endometrioid carcinomas and one carcinosarcoma. All four major histological types of ovarian carcinoma were represented [17]. The histological distribution was consistent with that previously reported for ovarian and uterine tumors of Japanese patients [18, 19].

### Germline mutations

**Figure 1:**
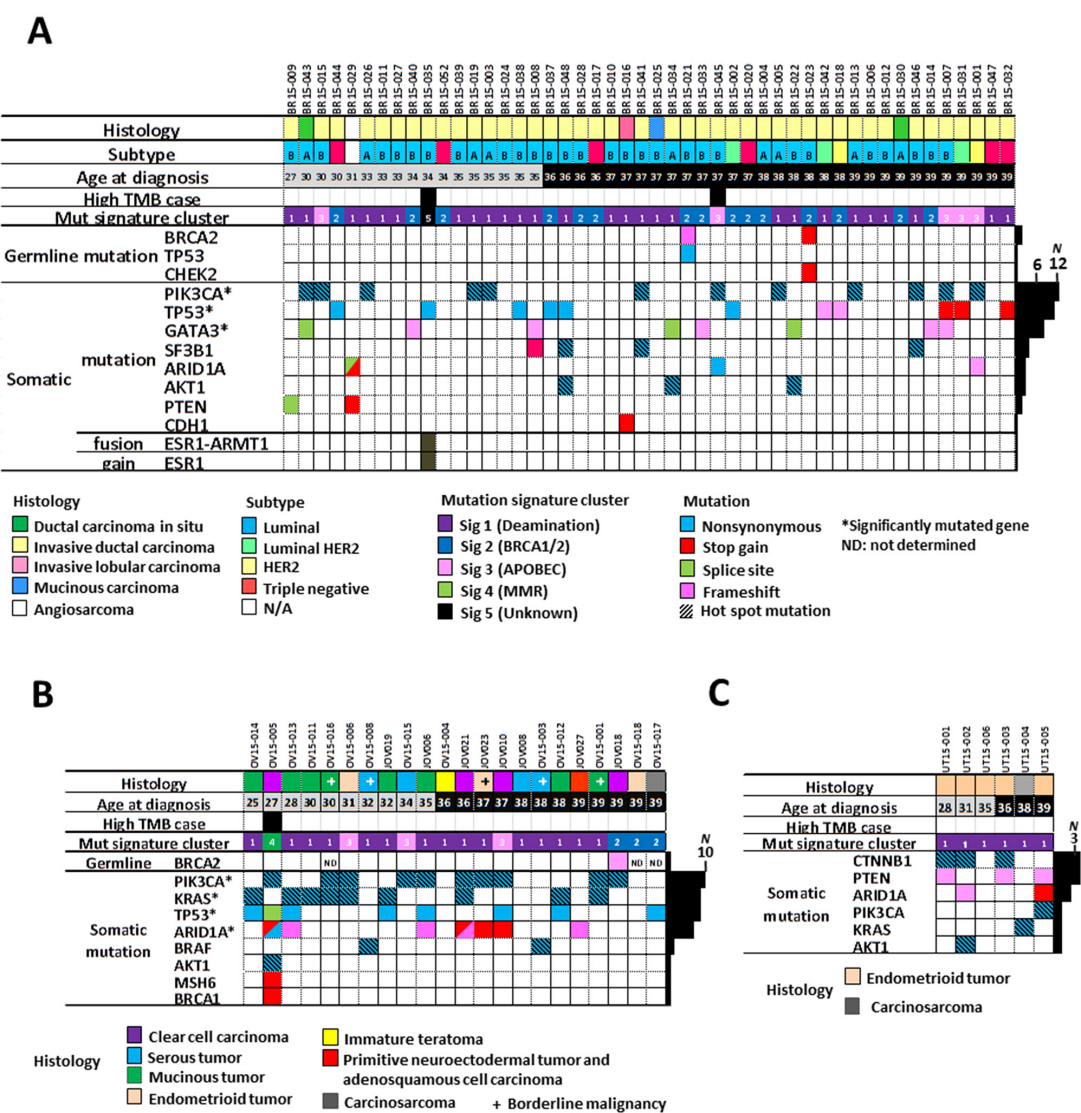
Gene aberration profiles of tumors of AYA Japanese females **(A)** Forty-eight breast, **(B)** 22 ovarian, and **(C)** six uterine tumors. Clinical and histological factors, tumor mutation burden, mutational signature cluster groups (see Figure 2), germline mutations in 25 cancer susceptibility genes [20], and somatic mutations in representative cancer census genes known to be aberrant in female tumors are shown. High tumor burden cases were defined as those with > 10 single nucleotide variants per Mb [21]. A/B subtypes of luminal type tumors are indicated by the characters A and B. ND, not determined; N/A, not applicable; ^*^, significantly mutated genes in breast and ovarian tumors defined by the MutSigCV program.

Exome sequencing data generated from non-tumor DNA from 73/76 cases (three ovarian tumor cases without informed consent for germline mutation analysis were excluded) were analyzed to identify germline mutations in 25 known cancer susceptibility genes [20]. Germline mutations were identified in three cases: 2/48 cases with breast (4.2%) and 1/19 cases with ovarian (5.3%) cancer carried pathogenic deleterious germline mutations in the *BRCA2* gene. The two cases with breast tumors also each carried an additional deleterious germline mutation: one in *TP53* and the other in *CHEK2*. No other cases showed germline mutations in the 25 genes tested (Figure 1, Supplementary Table 1).

### Somatic mutations

Next, we searched for somatic mutations by examining exome sequencing data from tumors from all 76 cases. A high tumor mutation burden (TMB), recently defined as > 10 SNVs/Mb [21], was found in two breast carcinomas (BR15-035T, TMB = 50.4 and BR15-045T, TMB = 16.4) and an ovarian carcinoma (OV15-005T, TMB = 250.9). Deleterious germline and somatic mutations, *i.e*., nonsense and frameshift insertion/deletion (indel) alterations, in six hypermutator genes consisting of four mismatch repair (MMR) genes (*MLH1*, *MSH2*, *MSH6*, and *PMS2*) and two DNA polymerase genes with proofreading function, *POLD* and *POLE*, were examined as potentially responsible for high TMB, since their aberration is established as associated with high TMB in a variety of human cancers [21-24]. The case with ovarian cancer had a deleterious somatic mutation in *MSH6*, a MMR gene, while the two cases with breast cancer lacked mutations in the six genes (Figure 1, Supplementary Table 1). The TMBs of the remaining breast and ovarian, and all six uterine, tumors were similar (medians 0.60, 0.64, and 0.59, respectively; *P* > 0.05 by Kruskal-Wallis test).

**Figure 2:**
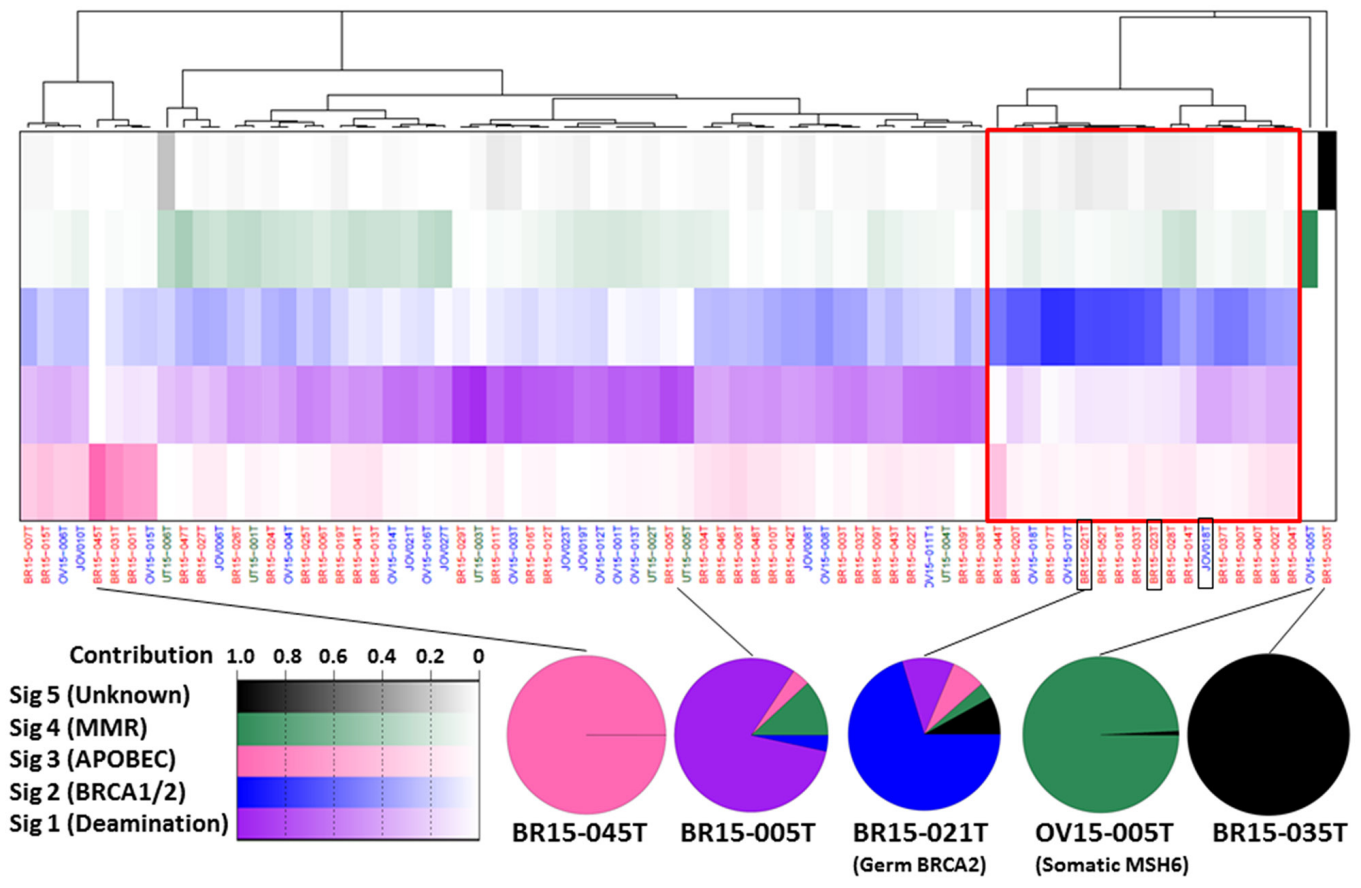
The spectrum of mutational signatures within 76 AYA female tumors Samples are ordered according to hierarchical clustering performed on signatures. The IDs of the three cases with germline *BRCA2* mutations are boxed in black. The top panel shows the proportion of each signature in each sample (increasing with color intensity as shown bottom left). The proportion of each signature in representative cases is represented bottom right.

The 76 tumors exhibited five mutational signatures, four of which showed high cosine similarity (≥ 0.9) with 30 known signatures deposited in the Catalogue of Somatic Mutations in Cancer (COSMIC) database (http://cancer.sanger.ac.uk/cosmic/signatures) and had been detected in breast, ovarian, and uterine cancer genomes in previous studies [12, 25] (Supplementary Figure 1). Hierarchical cluster analysis of mutational signatures revealed that the 76 cases could be divided into five groups (Figure 2). The largest group (*N* = 48; 63%) comprised cases in which COSMIC-signature 1 (resulting from spontaneous deamination of 5-methylcytosine) was predominant. In the second largest group (*N* = 18; 24%) COSMIC-signature 3 (associated with *BRCA1* and *BRCA2* mutations) was predominant; all three cases with germline *BRCA2* mutations were included in this group. In the third largest group (*N* = 8; 11%) COSMIC-signature 2 (attributed to activity of members of the APOBEC cytidine deaminase family) was predominant; however, expression levels of APOBEC genes were similar between these cases and those in other COSMIC signature groups (Supplementary Figure 1E). The remaining two groups each contained a single high TMB case. Case OV15-005T with the somatic *MSH6* mutation showed strong identity with the COSMIC-signature 6 cluster associated with MMR deficiency. BR15-035T showed strong identity with an unknown signature pattern enriched in CpC to CpA mutations.

### Profiles of aberrations in cancer gene census genes

MutSigCV analysis identified several Cancer Gene Census (CGC) genes (http://cancer.sanger.ac.uk/census/) as having significant roles in the development/progression of AYA tumors; *PIK3CA* and *TP53* were prominent in breast tumors, and *PIK3CA*, *KRAS*, *TP53*, and *ARID1A* in ovarian tumors (Figure 1), all of which are also frequently mutated in non-AYA breast and ovarian tumors [12, 16, 26-28]. A deleterious mutation in *CDH1* was identified in the invasive lobular carcinoma (ILC), BR15-016T, consistent with a previous study showing frequent *CDH1* mutation in ILC [29]. Among the ovarian tumors, *KRAS* mutations were more frequent in mucinous tumors than in other types (7/8 *vs.* 2/14; *P* = 0.0015 by Fisher’s exact test). *PIK3CA* and *ARID1A* mutations were more frequent in clear cell or endometrioid tumors than in other types (6/7 *vs.* 4/15 and 4/7 *vs.* 3/15; *P* = 0.016 and 0.11, respectively), consistent with previous studies of all-age-group ovarian tumors [13, 15, 27]. Mutations in *CTNNB1*, *PTEN*, and *ARID1A* were recurrent among the six uterine tumors (50%, 50%, and 33%, respectively), which is also consistent with previous reports from all-age-group uterine tumors [30]. These findings indicate that aberrations in the same sets of genes contribute to breast, ovarian, and uterine tumorigenesis in both AYA and non-AYA individuals.

RNA sequencing detected a novel in-frame fusion gene, *ESR1-ARMT1*, between *ESR1*, encoding estrogen receptor 1, and *ARMT1*, encoding acidic residue methyltransferase 1, in an ER+ luminal type invasive ductal carcinoma, case BR15-035T (Supplementary Figure 2). The increased genome copy numbers of both loci in this case, together with the location of these two genes neighboring *CCDC170* on chromosome 6q25, suggest that this fusion was generated by tandem duplication of the *ARMT1-CCDC170-ESR1* locus, as identified in breast cancers bearing the recurrent *ESR1-CCDC170* fusion [31].

### Proportion of cases with actionable gene aberrations

**Figure 3:**
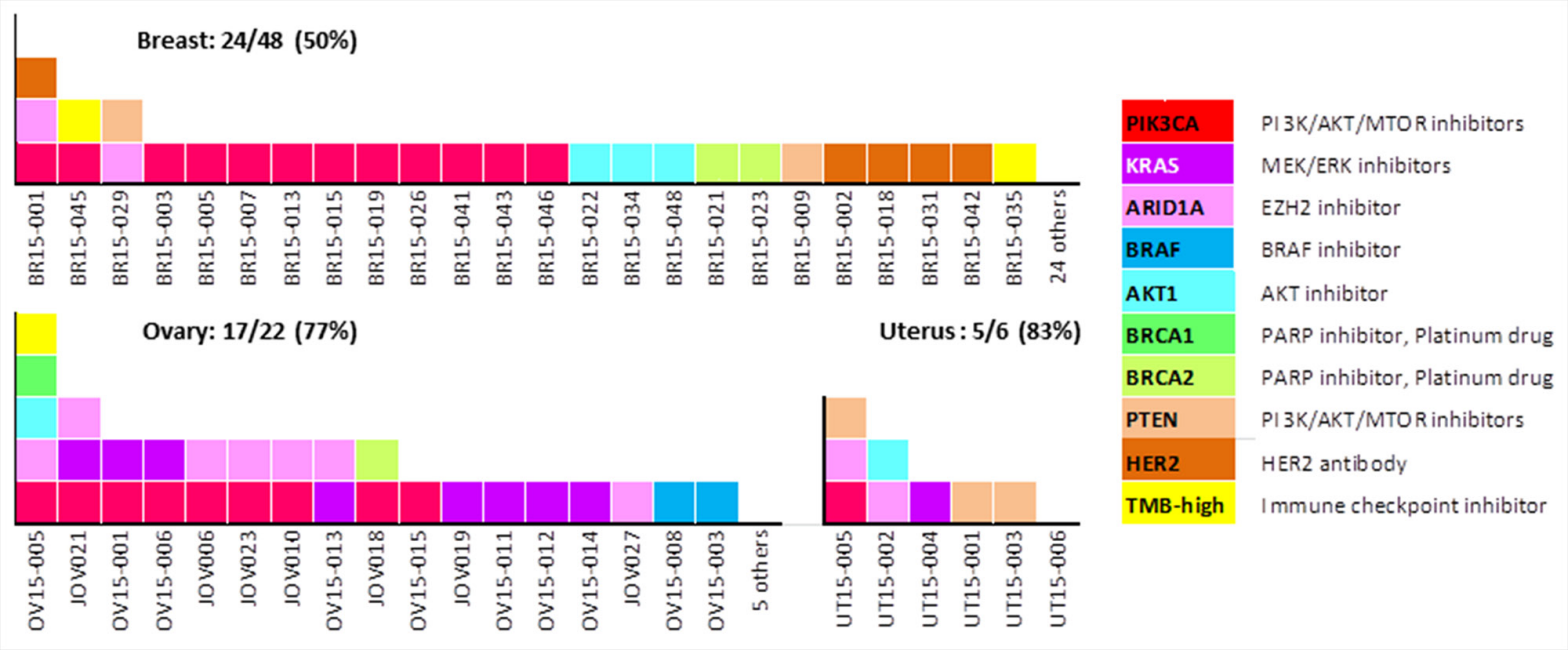
Fractions of AYA female tumors with potentially actionable gene aberrations Gene aberrations and their corresponding therapeutic agents are shown for each case.

Hot spot activating mutations in the *PIK3CA*, *KRAS*, *BRAF*, and *AKT1* genes, copy number gains in *HER2*, and deleterious *BRCA1*, *BRCA2*, *PTEN*, and *ARID1A* mutations were considered actionable gene aberrations (Figure 3), since drugs targeting the molecules encoded by these loci are available or being developed in clinical trials. In addition, high TMB was deemed an actionable aberration, as this feature is associated with response to immune checkpoint inhibitory therapy [21, 32]. Based on these criteria, 24 breast (50%), 17 ovarian (77%), and five uterine (83%) tumor cases were judged to have actionable gene aberrations.

## DISCUSSION

Here, the genome-wide profiles of 76 sporadic tumors of AYA Japanese females were investigated to determine the underlying mutagenic processes and elucidate actionable gene aberrations. Germline mutations in 25 genes established as involved in hereditary tumors [20] were detected only in a small fraction of patients: 4.2% of cases with breast tumors and 5.3% of those with ovarian tumors. The frequencies among breast and ovarian tumor cases were considerably lower than those found in sporadic cases among AYAs in the US (Supplementary Table 2). Thus, germline mutations in those susceptibility genes may contribute to the development of a smaller subset of sporadic AYA tumors in females in Japan than in those in the US. It was noted that two individuals with breast cancer with germline *BRCA2* mutation also carried another deleterious germline mutation, respectively. Double germline mutations have been observed in several US patients (Supplementary Table 2), therefore, their pathogenic and clinical significance in AYA-tumors should be further investigated in larger sets of samples.

Mutational signature analysis revealed that our cases had the same signatures as those observed in all-age-group studies of breast, ovarian, and uterine tumors [11-13]. The prevalence of COSMIC-signatures 1 and 2 in female AYA tumors was in common with that in a variety of tumors [11], indicating that the mutagenic mechanisms are similar [11]. By contrast, a substantial proportion of the cases (24%) bore a signature of mutagenesis associated with *BRCA1* and *BRCA2* deficiency, which is exclusively observed in breast, ovarian, and pancreatic tumors [11]. This mutational signature profile of AYA tumors is quite similar to that of sporadic breast and ovarian cancers in general [12, 13]. Therefore, the mutation burden during tumorigenesis is likely to be similar between tumors in AYA and non-AYA females. The breast cancer case, BR-035T, showed a signature highly enriched in CpC to CpA mutations. To the best of our knowledge, this patient had not suffered from specific carcinogen exposure; therefore, the underlying process that initiated mutagenesis remains unclear.

Consistent with the mutational signature data, AYA tumors showed mutations in the same set of genes as non-AYA tumors. Deleterious mutations in *GATA3*, an activating *SF3B1* mutation (K700E), and an activating *AKT1* mutation (E17K) were observed in eight (16.7%), three (6.3%), and three (6.3%) breast tumors, respectively. These frequencies are higher than those in overall breast cancers [12, 16, 33]. Thus, these gene aberrations could be preferentially involved in the development of tumors in AYA females. In the present study, significant fractions of breast (50%), ovarian (77%), and uterine (83%) tumors had actionable gene mutations and gains (Figure 3), while actionable oncogene fusions, as frequently observed in AYA lung tumors [34, 35], were not discovered. Recently, it was reported that a mutational “BRCAness” signature is a predictor for functional *BRCA1/BRCA2* deficiency [36]; therefore, the 15 *BRCA1/2* mutation-negative breast/ovarian tumors with a predominant COSMIC-signature 3 could be responsive to PARP inhibitors and platinum agents due to deficiency in DNA double strand break repair. If that were the case, the fractions of breast and ovarian tumors with actionable gene aberrations in the current study would increase to 74% and 86%, respectively (Supplementary Figure 3).

An *ESR1-ARMT1* fusion was detected in a case of breast carcinoma. Several types of gene fusions including *ESR1* have been reported in ER+ breast cancers [37]. All known ESR fusion proteins have a common structure, where the ligand-binding domain of the ESR1 protein is absent, but the hormone-independent transactivation domain and DNA-binding domain are retained, suggesting their significance in resistance to endocrine therapy. The ESR1-ARMT1 fusion protein identified in the current study retains the ligand-binding and transactivation domain, but lacks the DNA-binding domain (Supplementary Figure 2A), and patient BR15-035T, from whom it was isolated, had not received endocrine therapy. Thus, the effect of *ESR1-ARMT1* fusion on resistance to endocrine therapy is unknown.

Overall, our results from gene profiling of tumors from 76 female AYAs lead us to conclude that the mutational processes in these malignancies, as well as their aberrant genes, are largely shared with non-AYA tumors. High frequencies of actionable gene aberrations, including high TMB and the “BRCAness” mutational signature, indicate that the efficacy of molecular targeting and immune checkpoint inhibitory therapies should be studied in AYA patients, along with non-AYA patients. In addition, more extensive study of germline mutations in genes other than the 25 examined in this study will facilitate our understanding of hereditary factors involved in AYA tumor development.

## MATERIALS AND METHODS

### Patients

The AYA tumors analyzed in the present study were obtained from consecutive cases aged 15 to 39 years, who were diagnosed with breast, ovarian, or uterine tumors and underwent surgery at the National Cancer Center Hospital (NCCH), Tokyo, Japan, or at the Jikei University Hospital (JUH), Tokyo, Japan, and for whom snap-frozen tumor and non-tumor tissues were available in the NCCH and JUH-Gynecology Biobanks. None of the 76 patients had received any pre-surgical treatment, and there was no obvious family history of cancers. Seventy-three patients provided informed consent for both somatic and germline gene aberration analysis. The remaining three, OV15-016, OV15-017, and OV15-018, only provided informed consent for somatic mutation analysis, in which non-tumor DNA is used as a reference to identify somatic mutations in tumor DNA; these three patients did not give consent for germline mutation analysis, where non-tumor tissue DNA is used for detection of germline mutations. The institutional review boards of the National Cancer Center and Jikei University approved the study. Clinical data for each patient, including their age, and tumor stage, histology, and subtype, were collected retrospectively. Breast tumors were diagnosed according to the Union for International Cancer Control TNM classification (7th edition). Subtyping of breast tumors was performed based on the status of ER, PgR, HER2, and Ki67, which were examined by immunohistochemistry, as previously described [19]. Ovarian and uterine tumors were diagnosed in accordance with the International Federation of Gynecology and Obstetrics (FIGO) system and classified according to the World Health Organization (WHO) classification of tumors [38].

### Whole exome and RNA sequencing

Exome sequencing was conducted using 200 ng of genomic DNA isolated from snap-frozen tumor and non-tumor tissues obtained from 76 patients. Exome capture was performed using the Agilent SureSelect Human V5 platform, according to the manufacturer’s instructions. The median sequencing depths of tumor and non-tumor DNA were 203 (range, 128–228) and 104 (79–136), respectively. Somatic single nucleotide variants (SNVs) were called using the MuTect program for variants present in bi-directional reads [39]. Somatic insertion/deletion (indel) mutations were called using the GATK Somatic Indel Detector, while germline SNVs and indels were called using the GATK program (https://www.broadinstitute.org/gatk/). Significantly mutated genes were defined by a *q* value of < 0.10, using the MutSigCV program [28]. Pathogenic germline mutations in 25 known cancer susceptibility genes [20] were defined as “pathogenic variants” deposited in the ClinVar database (http://www.ncbi.nlm.nih.gov/clinvar/), and as deleterious variations, *i.e*., nonsense SNVs and frameshift indel variants. The 25 genes examined consisted of 12 breast cancer susceptibility genes (*ATM*, *BARD1*, *BRCA1*, *BRCA2*, *BRIP1*, *CDH1*, *CHEK2*, *NBN*, *PALB2*, *PTEN*, *STK11*, and *TP53*) and 13 cancer susceptibility genes (*APC*, *BMPR1A*, *CDK4*, *CDKN2A*, *EPCAM*, *MLH1*, *MSH2*, *MSH6*, *MUTYH*, *PMS2*, *RAD51C*, *RAD51D*, and *SMAD4*). These genes were selected because they are well-documented moderate- and high-risk genes for female tumors [20].

RNA samples (200 ng) extracted from snap-frozen tissues using TRizol reagent (Thermo Fisher Scientific) were subjected to RNA sequencing using the TruSeq RNA Sample Prep Kit (Illumina). Fusion transcripts were detected using the TopHat-Fusion algorithm [40].

### Mutational signature analysis

Mutational signatures were analyzed by non-negative matrix factorization (NMF), which was applied to the 96 possible mutations occurring in a trinucleotide context, as previously described [11, 25]. NMF was performed with various numbers of signatures, from one to ten, in this study. Obtained signatures were compared with those in the COSMIC database (http://cancer.sanger.ac.uk/cosmic/siganatures). The similarity was quantified using cosine similarity as previously described [11, 25].

### Statistical analyses

Statistical analyses of differences in clinico-pathological factors and genetic aberrations were tested by Mann-Whitney U, Kruskal-Wallis, Pearson’s chi^2^, and Fisher’s exact tests.

### Further information

See Supplementary Materials.

## SUPPLEMENTARY MATERIALS FIGURES AND TABLES



## Abbreviations

AYA: adolescent and young adult
CGC: Cancer Gene Census
COSMIC: Catalogue of Somatic Mutations in Cancer
ER: estrogen receptor
HER2: human epidermal growth factor receptor 2
indel: insertion/deletion
ILC: invasive lobular carcinoma
JUH: Jikei University Hospital
MMR: mismatch repair
NCCH: National Cancer Center Hospital
NMF: non-negative matrix factorization
PgR: progesterone receptor
SNV: single nucleotide variant
TMB: tumor mutation burden
