# Supplementary material for: Gene aberration profile of tumors of adolescent and young adult females
Source: Oncotarget. 2017 Dec 29;9(5):6228–37. doi: 10.18632/oncotarget.23765 (PMC5814207; doi:10.18632/oncotarget.23765)
Supplement: Supplementary file 1 [file oncotarget-09-6228-s001.pdf]

Gene aberration profile of tumors of adolescent and young adult females

SUPPLEMENTARY MATERIALS

Patients and subtyping of breast tumors

National Cancer Center Hospital cases were a series of patients with sporadic breast (BR; $N = 48$), ovarian (OV; $N = 14$), or uterine (UT; $N = 6$) tumors diagnosed between the ages of 15 and 39 years, who underwent surgery between 2011 and 2015. Jikei University Hospital cases were a series of patients with sporadic ovarian tumors ($N = 8$) diagnosed between the ages of 15 and 39 years who underwent surgery between 2003 and 2015.

Subtyping of breast tumors was performed based on the status of estrogen receptor (ER), progesterone receptor (PgR), human epidermal growth factor receptor 2 (HER2), and Ki67, which were examined by immunohistochemistry (IHC), as previously described [1]. Primary antibodies used for IHC were mouse monoclonal anti-ER (clone 1D5; Dako, Glostrup, Denmark), mouse monoclonal anti-PgR (clone PgR636; Dako), and rabbit polyclonal anti-HER2 (HercepTest II; Dako).

Whole exome sequencing

Exome sequencing was performed on the Illumina HiSeq 2500 platform using 150 bp paired-end reads (Illumina). Basic alignment and sequence quality control were conducted using the Picard (http://picard.sourceforge.net/) and Firehose (http://www.broadinstitute.org/cancer/cga/firehose) pipelines. Reads were aligned against the UCSC reference human genome (Hg19) using the Burrows Wheeler Aligner Multi-Vision software package (http://bio-bwa.sourceforge.net/). As duplicate reads were generated during the PCR amplification process, paired-end reads that aligned to the same genomic positions were removed using SAMtools (http://samtools.sourceforge.net/).

Somatic single nucleotide variants (SNVs) were called using the MuTect program, which applies a Bayesian classifier to allow the detection of somatic mutations with low allele frequencies [2]. Somatic insertion/deletion (indel) mutations were called using the GATK Somatic Indel Detector, while germline SNVs and indels were called using the GATK program (https://www.broadinstitute.org/gatk/). Somatic and germline SNV and indel detection was corroborated by visual examination using the Integrative Genomics Viewer software (http://www.broadinstitute.org/igv/).

Significantly mutated genes were defined by a q value of < 0.10 using the MutSigCV program [3]. This analysis was performed for breast and ovarian tumors, but not uterine tumors, due to the small number of samples. Cancer Gene Census (CGC) genes were those in the COSMIC v70 database (https://cancer.sanger.ac.uk/cancergenome/projects/cosmic/).

RNA sequencing and copy number examination

RNA was extracted from snap-frozen tumor tissues using TRizol reagent (Thermo Fisher Scientific). The quality and quantity of the RNA was examined using a Bioanalyzer (Agilent). RNA samples from 72 patients had RNA integrity numbers > 6.0, and were therefore suitable for sequencing. The TruSeq RNA Sample Prep Kit (Illumina) was used to prepare RNA sequencing libraries from 200 ng of total RNA. The resultant libraries were subjected to paired-end sequencing of 150 bp reads on a HiSeq 2500 system (Illumina). Fusion transcripts were detected using the TopHat-Fusion algorithm [4].

Genome copy number alterations associated with ESR1 fusion were examined by quantitative real-time PCR (qRT-PCR). qRT-PCR was performed using a TaqMan Copy Number Assay (Thermo Fisher Scientific, Waltham, MA, USA) and the 7900 HT Fast Real-Time PCR system (Thermo Fisher Scientific). DNA (10 ng) was added to each 10 μL PCR reaction containing TaqMan Universal Genotyping Master Mix. All assays were performed in triplicate or quadruplicate. The following TaqMan probes were purchased from Thermo Fisher Scientific: ESR1-exon 5 (Hs01325837_cn), CCDC170 (Hs01144992_cn), ARMT1-intron 3 (Hs03592242_cn), ARID1B (Hs03581035_cn), EZR (Hs02997852_cn), and LATS1 (Hs02117479_cn). RNase P (#4403326) was used as a reference control. Predicted copy numbers were calculated using CopyCaller v2.0 (Thermo Fisher Scientific) and normalized to the mean of non-tumor breast tissues of four patients: BR15-005, BR15-010, BR15-015, and BR15-020. A calculated copy number ≥ 5 was deemed a copy number gain.

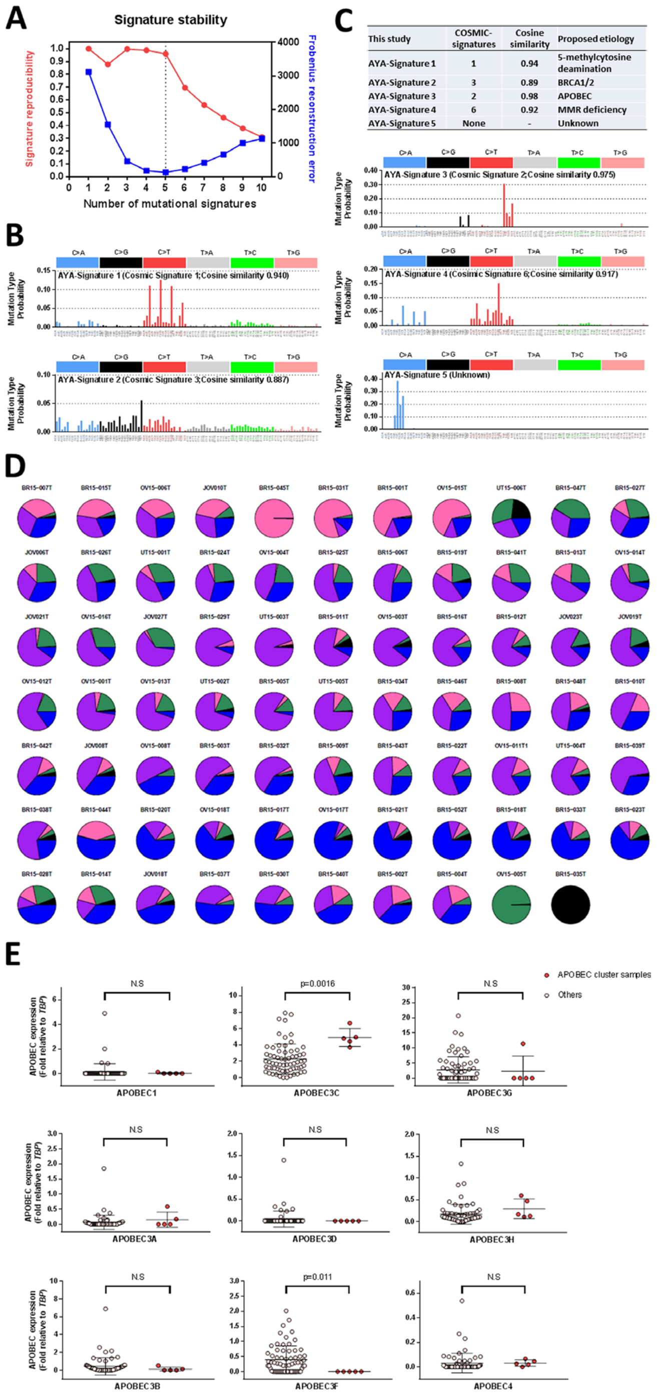

Supplementary Figure 1: Mutational signature analysis. **(A)** Signature stability according to number of signatures. Signature reproducibility (red) and Frobenius reconstruction error (blue) by non-negative-matrix factorization (NMF) analysis. NMF analysis was performed against various numbers of signatures, from one to ten (*x*-axis). Five mutational signatures were estimated as optimal because at this point the signature stability remained high and the reconstruction error was low (indicated by the dotted line). **(B)** Five mutational signatures detected in 76 AYA female tumors. **(C)** Cosine similarity between each of the five signatures from this analysis and 30 known signatures (http://cancer.sanger.ac.uk/cosmic/signatures). AYA-signature 5 did not show a high similarity (of >0.6) to any of the 30 COSMIC signatures. **(D)** Mutational signature profiles of each of the 76 tumors. Fractions of mutations corresponding to AYA-signatures 1–5 are shown by purple, blue, pink, green and black, respectively. The cases are ordered according to clustering data shown in Figure 2. **(E)** Expression levels of APOBEC genes by APOBEC mutation cluster. Expression levels are shown by FPKM fold values relative to TBP as in a previous study [38]. Difference in expression level was examined by the Mann-Whitney U test. N.S., not statistically significant.

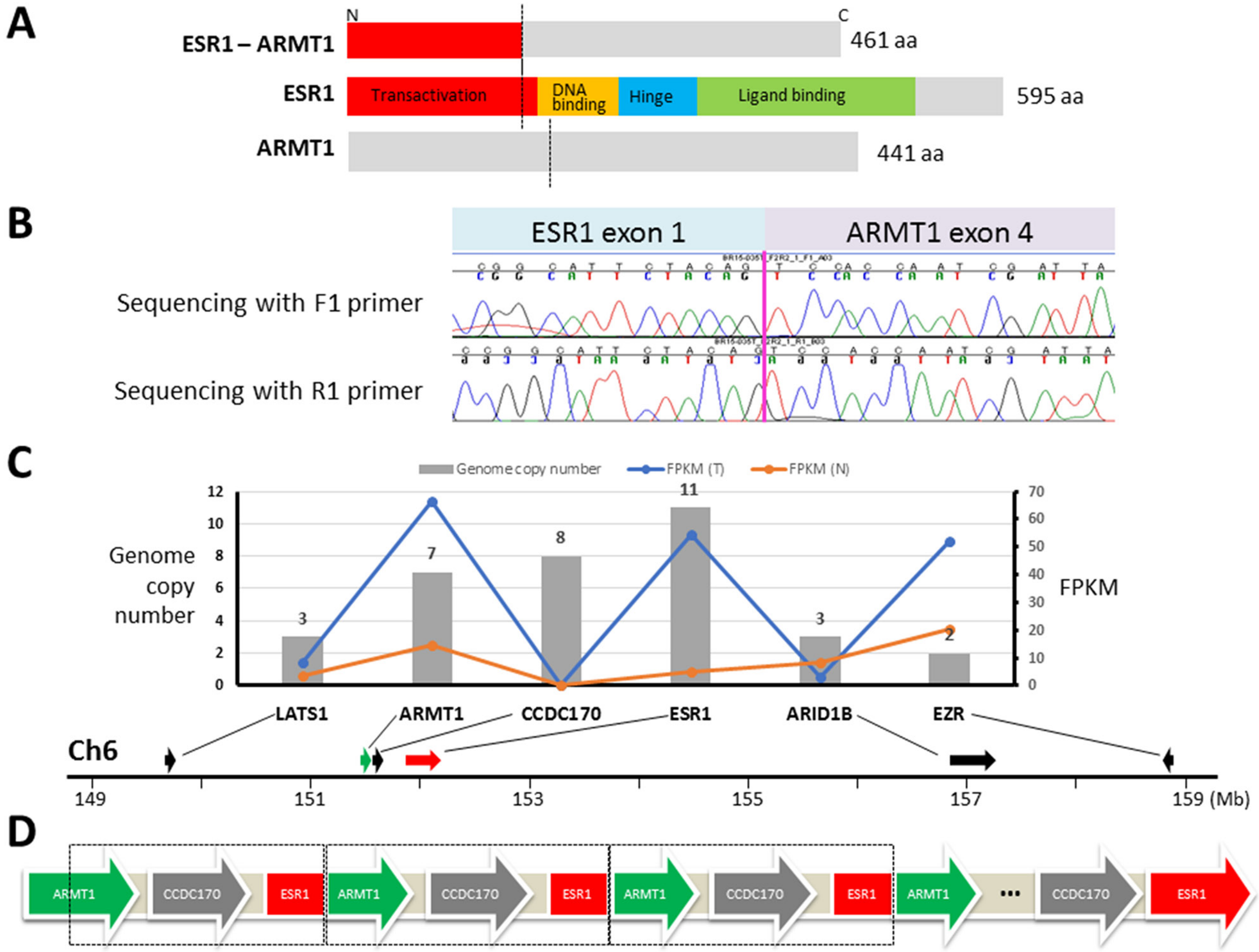

Supplementary Figure 2: *ESR1-ARMT1* fusion detected in case BR15-035T. (A) Schematic representation of the ESR1-ARMT1 fusion protein with the wild type ESR1 and ARMT1. **(B)** Sanger sequencing of RT-PCR product for the *ESR1-ARMT1* fusion from the BR15-035T tumor. Complementary DNA from BR15-035T tumor RNA was subjected to PCR using a ESR1 primer (5'-ATGACCATGACCCTCCACAC-3') and a ARMT1 primer (5'-CAAAGGGGACCATCGTACTG-3'). RT-PCR amplicons were subjected to Sanger sequencing in forward (top panel) and reverse directions (bottom panel) using a forward (5'-CAGGTGCCCTACTACCTGGA-3') and a reverse (5'-GCAATGATGGATTCCTGTGA-3') primers, respectively. **(C)** Focal amplification at the *ARMT1* and *ESR1* loci in BR15-035T associated with elevated expression. Genome copy number examined by quantitative real-time PCR of genomic DNAs and expression levels (FPKM values) obtained by RNA sequencing are shown. **(D)** Tandem duplication as a potential genetic mechanism generating *ESR1-ARMT1* fusion.

Supplementary Table 1: AYA tumor cases with germline mutations

Sample	Age at diagnosis	Tumor site	Subtype	Histology	Stage	Germline mutation 1	Germline mutation 2
BR15-021T	37	Breast	Luminal B	IDC	2a	*BRCA2* (c.8588dupA: p.E2863fs)	*TP53* (c.C170T: p.A57V)
BR15-023T	38	Breast	Luminal B	IDC	3a	*BRCA2* (c.C6952T: p.R2318X)	*CHEK2* (c.C283T: p.R95X)
JOV018T	39	Ovary		Clear cell	2a	*BRCA2* (c.6445_6449del: p.I2149fs)	

IDC, invasive ductal carcinoma.

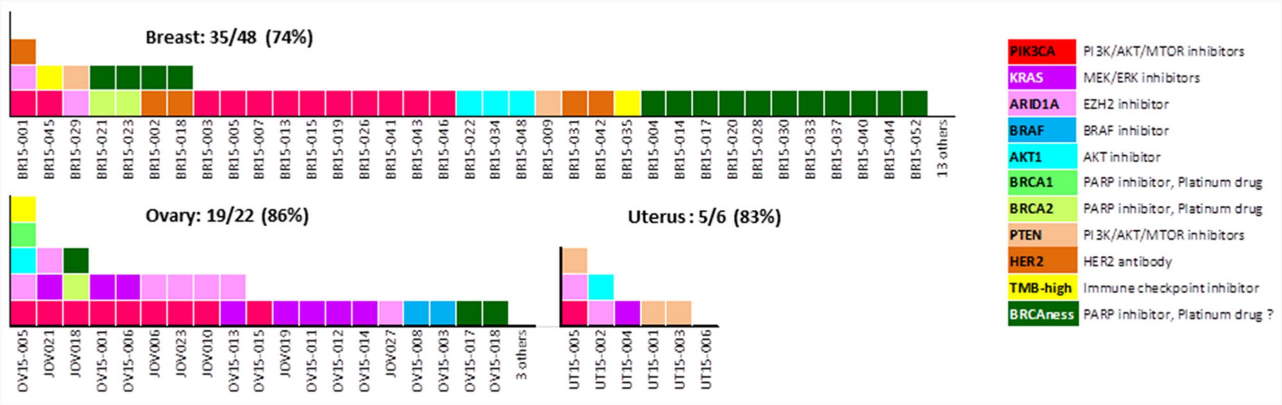

Supplementary Figure 3: Fractions of AYA female tumors with potentially druggable gene aberrations. Gene aberrations and therapeutic agents matched to them are shown for each case. Mutational "BRCAness" signature is considered here as a predictor for functional *BRCA1/BRCA2* deficiency.

Supplementary Table 2: Germline mutations in AYA female tumors

	Japan (this study)			US[a]	US[b]	US[c]
	**Breast**	**Ovary**	**Uterus**	**Breast**	**Breast**	**Ovary**
No. of samples	48	19	6	70	180	235
Age (y)	27–39	25–39	28–39	26–39	26–45	23–92
No. of mut (+) samples	2	1	0	25	30	28–32
%	4.2%	5.3%	0.0%	36%	17%	12–14%
BRCA1				13	15	12–22
BRCA2	2	1		8	7	5-11 (1: *BRCA1*)
ATM					3 (1: *BRCA2*)	NT
ATR				1 (*BRCA2*)		NT
BRIP1				1	1	NT
CHEK2	1 (*BRCA2*)			1	4 (1: *ATM*)	NT
NBN						NT
PTEN				1		NT
RAD50				1		NT
ATM						NT
CDH1						NT
PMS2					1 (*BRCA1*)	NT
TP53	1 (*BRCA2*)					NT
PALB2					1	NT
MSH6						NT
RAD51C						NT
RAD51D					1	NT

[a]Serena *et al.*, Nature, 2016; [b]Nadine *et al.*, J Clin Oncol, 2016; [c]Hennessy *et al.*, JCO, 2010; mut, mutation; NT, not tested.

REFERENCES

1. Akahira J, Tokunaga H, Toyoshima M, Takano T, Nagase S, Yoshinaga K, Tase T, Wada Y, Ito K, Niikura H, Yamada H, Sato A, Sasano H, Yaegashi N. Prognoses and prognostic factors of carcinosarcoma, endometrial stromal sarcoma and uterine leiomyosarcoma: a comparison with uterine endometrial adenocarcinoma. Oncology. 2006; 71: 333-340.
2. Cibulskis K, Lawrence MS, Carter SL, Sivachenko A, Jaffe D, Sougnez C, Gabriel S, Meyerson M, Lander ES, Getz G. Sensitive detection of somatic point mutations in impure and heterogeneous cancer samples. Nat Biotechnol. 2013; 31: 213-219.
3. Lawrence MS, Stojanov P, Polak P, Kryukov GV, Cibulskis K, Sivachenko A, Carter SL, Stewart C, Mermel CH, Roberts SA, Kiezun A, Hammerman PS, McKenna A, et al. Mutational heterogeneity in cancer and the search for new cancer-associated genes. Nature. 2013; 499: 214-218.
4. Kim D, Salzberg SL. TopHat-Fusion: an algorithm for discovery of novel fusion transcripts. Genome Biol. 2011; 12: R72.
